# Characterization of the complete chloroplast genome of ornamental plant, *Bougainvillea peruviana* (Nyctaginaceae)

**DOI:** 10.1080/23802359.2020.1768948

**Published:** 2020-08-28

**Authors:** Guofeng Liu, Shiou Yih Lee, Xing Hu, Miaomiao Sun, Jianzhong Ni, Wei Wang, Seping Dai, Lin Ruan

**Affiliations:** a Guangzhou Institute of Forestry and Landscape Architecture, Guangzhou, China; b State Key Laboratory of Biocontrol and Guangdong Provincial Key Laboratory of Plant Resources, School of Life Sciences, Sun Yat-sen University, Guangzhou, China

**Keywords:** *Bougainvillea*, chloroplast genome, ornamental, landscaping, Illumina sequencing

## Abstract

*Bougainvillea peruviana* is a widely domesticated ornamental plant species. However, studies on *B. peruviana* are limited. In this study, we reported the complete chloroplast (cp) genome of *B. peruviana*. The cp genome is 154,465 bp in length, containing a large single-copy (LSC) of 85,563 bp and a small single-copy (SSC) of 18,050 bp, which are separated by a pair of inverted repeats (IRs) of 25,426 bp, each. A total of 132 genes, including 86 protein-coding genes, 38 tRNA genes, and eight rRNA genes, were predicted. The overall GC content for the cp genome is 36.5%. The maximum-likelihood tree constructed based on cp genome sequences showed that *B. peruviana* is placed under Nyctaginaceae and is diverged before *Bougainvillea glabra* and *Bougainvillea spectabilis* under strong bootstrap support.

*Bougainvillea* is a versatile and remarkable ornamental plant widely used in the gardens (Tripathi et al. 2017). Among all *Bougainvillea* species, *Bougainvillea peruviana*, which is originated from Peru, is regarded as one of the major *Bouganvillea* species responsible for the vast applications in urban landscaping (Salam et al. 2017). Due to its attractive and colorful bracts, many crosses among various species have produced new hybrid species and important cultivars, wherein hybrids such as *Bougainvillea* × *buttiana* and *Bougainvillea* × *spectoperuviana* are common *Bougainvillea* hybrid species derived from *B. peruviana* (Salam et al. 2017). Although the species has been domesticated in many countries, information on this species is scarce (Abarca-Vargas and Petricevich 2018). The commercial value of *B. peruviana* has overshadowed genetic studies on this important horticultural species. Therefore, in this study, we characterized the complete chloroplast (cp) genome sequence of *B. peruviana* using the next-generation sequencing technique.

DNA extraction was carried out using the fresh leaf samples collected from the *B. peruviana* planted in the Germplasm Resource Nursery of Ornamental Plants, Guangzhou Institute of Forestry and Landscape Architecture (GIFLA), Guangdong Province of China (N113°20′25′′, E23°13′47′′). The voucher specimen (voucher record number: GIFLA-Bope-2019-08-30) was deposited at the voucher collection room of GIFLA. A 300-bp insert size genomic library was constructed using TruSeq DNA Sample Prep Kit (Illumina, San Diego, CA) and sequencing was conducted on an Illumina Novaseq platform. Approximately 6 GB of raw data of 150-bp paired-end reads were generated and assembled using NOVOPlasty (Dierckxsens et al. 2017), using the *rbcL* gene sequence of *Bougainvillea glabra* (GenBank accession number: MG833637) as the seed sequence. Genome annotation was conducted using GeSeq (Tillich et al. 2017) and manually corrected.

The complete cp genome sequence of *B. peruviana* (GenBank accession number: MT407463) is 154,465 bp in length, containing a large single-copy (LSC) region (85,563 bp), small single-copy (SSC) region (18,050 bp), and a pair of IRs (25,426 bp, each). A total of 132 genes were predicted, including 86 protein-coding genes, 38 tRNAs, and 8 rRNAs. The total GC content of the cp genome is 36.5%.

Phylogenetic analysis was conducted using RAxML available in the CIPRESS Science Gateway web portal (Miller et al., 2010), using 1000 bootstrap replicates, based on the cp genome sequences of 10 species in the family Nyctaginaceae, and three species, *Petivera alliacea* (Petiveriaceae, MH286334), *Sarcobatus verniculatus* (Sarcobataceae; MH376309), and *Phytolacca insularis* (Phytolaccaceae; MH376309), were included as an outgroup. The maximum-likelihood tree showed that *B. peruviana* is clustered with other members from the family Nyctaginaceae, and is diverged before *Bougainvillea glabra* and *Bougainvillea spectabilis* under strong bootstrap support (100%; Figure 1).

**Figure 1.**
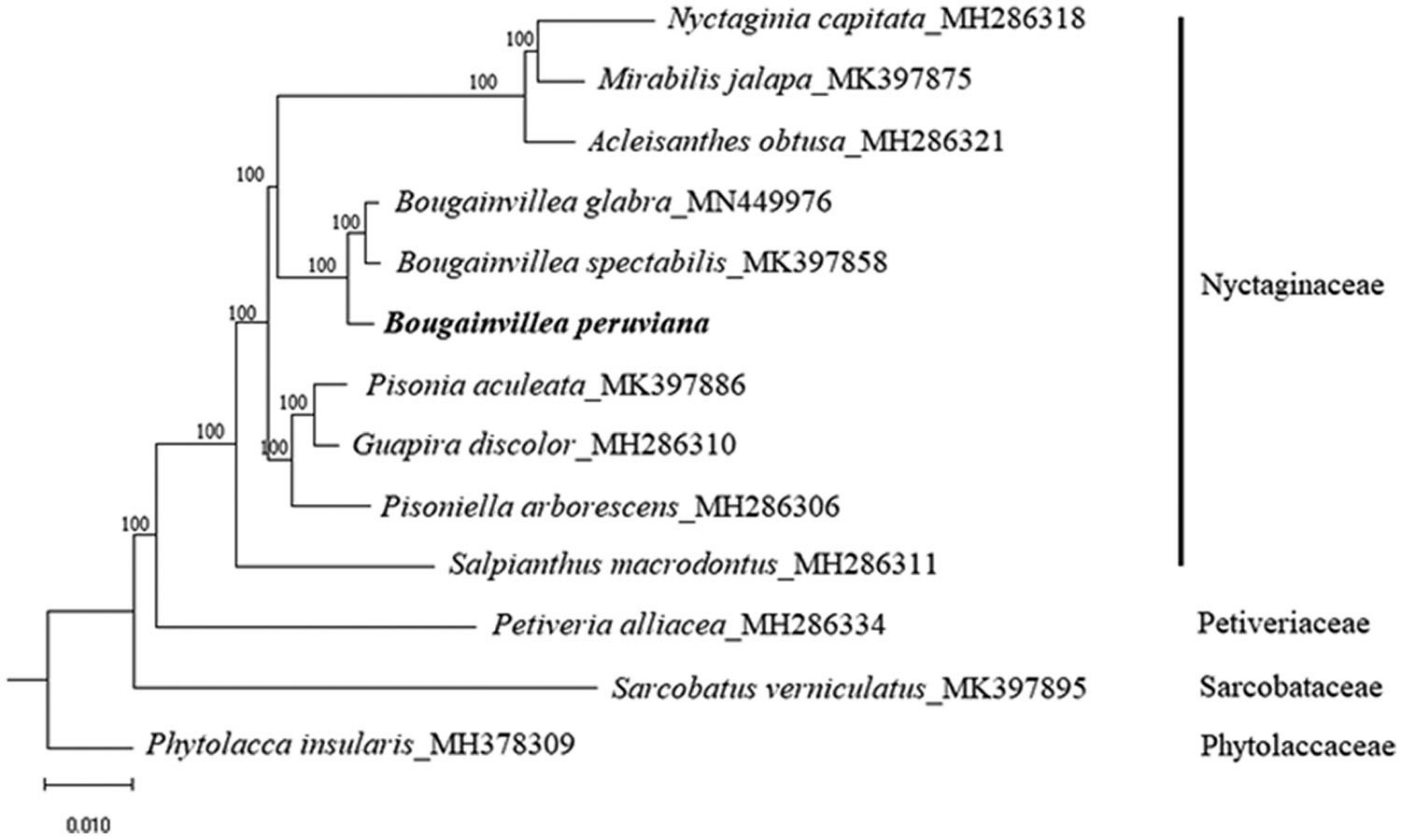
Maximum-likelihood tree based on the chloroplast genome sequences of 10 species from the family Nyctaginaceae, with *Petiveria alliacea* (Petiveriaceae), *Sarcobatus vermiculatus* (Sarcobataceae), and *Phytolacca insularis* (Phytolaccaceae) as outgroup. Shown next to the nodes are bootstrap support values based on 1000 replicates.

## Data Availability

The data that support the findings of this study are openly available in the NCBI GenBank at http://www.ncbi.nlm.nih.gov, accession number MT407463.
